# Effects of Selenium on Differentiation and Antioxidant Activity of Sclerotium of *Penicillium thomii* Q1 Strain

**DOI:** 10.1155/2020/2368245

**Published:** 2020-04-26

**Authors:** Wenjing Zhao, Feihong Zhai, Jianrong Han

**Affiliations:** ^1^Taiyuan Normal University, Jinzhong, China; ^2^Shanxi University, Taiyuan, China

## Abstract

Selenium is an essential trace element, which has certain antioxidant properties. Na_2_SeO_3_ is toxic, and its use is limited. SeMet, as an organic selenium, is less toxic than Na_2_SeO_3_. In this experiment, different concentrations of Na_2_SeO_3_ and SeMet were added to MEA and PDA media to observe the effect of selenium on the sclerotium differentiation of Q1 strain, and the contents of carotenoids, ascorbic acid, and total phenol and their reducing power, DPPH free radical scavenging ability, ferrous ion chelating ability, and superoxide anion scavenging ability were determined. Meanwhile, the orthogonal design was used to optimize the selenium enrichment culture conditions of Q1. The results showed that the addition of selenium in the PDA medium was not conducive to the differentiation of Q1 strain. The addition of inorganic and organic selenium in the MEA medium at different concentrations resulted in the accumulation of carotenoids, ascorbic acid, phenols, and selenium in the sclerotia of Q1 strain, and the contents of carotenoids, ascorbic acids, and selenium in the sclerotia of Q1 strain were increased to different degrees, but it cannot increase the content of total phenol. In addition, when the concentration of Na_2_SeO_3_ and SeMet in the medium was 10 *μ*g/mL, the reducing power of the extract was improved. The experimental results can provide a new research idea for the utilization and development of Penicillium sclerotium and selenium.

## 1. Introduction

Certain soil-borne fungi differentiate by forming compact multicellular structures called sclerotia in order to propagate asexually and survive in nature under adverse environmental conditions [1–4]. Because of the biological and agricultural significance of the sclerotiogenic fungi, considerable research efforts have been directed towards elucidating the mechanism of sclerotial biogenesis [5]. Oxidative stress is a disturbance of the cellular redox status that is often observed in stress situations. In nonstress conditions, ROS and other oxidants are balanced against the antioxidative defense system, which is composed of enzymes as well as metabolites [6]. All subcellular compartments contain specific antioxidative enzymes or metabolites that are coordinated and controlled by underlying regulatory mechanisms. Some nonenzymatic molecules can be extracted from sclerotia. These nonenzymatic molecules like ascorbic acid influence multiple signaling pathways [7] and can scavenge free radicals as natural antioxidants. Due to many synthetic antioxidants such as butylated hydroxyanisole, butylated hydroxytoluene, t-butylhydroquinone, and propyl gallate, there exist potential health hazards in many fields [8]. Currently, there is a great deal of interest in the study of natural compounds with free radical scavenging capacity. Researchers are continuously seeking those natural antioxidants that will sufficiently protect fats and oils from oxidation. Recent research has focused on antioxidant compounds derived from leaves and fruit of olive trees, numerous fruits and vegetables, and aromatic plants and spices [9]. But the antioxidant activity of fungi which produced sclerotia (mainly microsclerotia) has not been reported.

Selenium has many important biological functions: (1) antioxidant function [10]; (2) enhancing immune function. Selenium has significant effects on specific immunity (including cellular immunity and humoral immunity) and nonspecific immunity in the immune system [10–11]; (3) the function of antagonizing metal toxicity; (4) the function of promoting growth; and (5) other functions. Selenium also has important functions such as improving animal reproductive capacity, preventing cancer, and protecting the heart [11].

A strain of *Penicillium thomii* Q1 was isolated from a soil sample and was able to form abundant orange, sand-shaped sclerotia in which carotenoids accumulated. We had studied the culture condition influencing sclerotial growth and carotenoid production of Q1 strain [12].

In the body, selenium is an essential trace element, which has a certain degree of oxidation resistance, and it will have a synergistic effect with VE and other antioxidants [11]. Carotenoid and ascorbic acid can be accumulated in the sclerotia of Q1 strain, and they have certain antioxidant activity [13–14].

In this experiment, different concentrations of sodium selenite (Na_2_SeO_3_) and selenomethyline (SeMet) were added to MEA and PDA media to observe the effect of selenium on the sclerotium differentiation of Q1 strain, and the contents of carotenoids, ascorbic acid, and total phenol and their reducing power, DPPH free radical scavenging ability, ferrous ion chelating ability, and superoxide anion scavenging ability were determined.

## 2. Experimental

### 2.1. Strain

The strain of *Penicillium* sp. Q1 was isolated from the soil of Wutai Mountain in Shanxi Province and preserved in CA medium. Q1 strain was identified as *Penicillium thomii* (KC966729) by rDNA ITS sequence analysis [15].

### 2.2. Media

The media are the following.

*MEA*: malt extract 20 g, peptone 1.0 g, glucose 20 g, agar 20 g, and distilled water 1000 mL.

*Potato glucose agar medium (PDA)*: 1000 mL 20% potato juice, 20 g glucose, 18 g agar. Weigh 200 g potatoes, add 1000 mL water, boil for 20 minutes, and filter. Add enough water to 1000 mL in the filtrate, and the 20% potato juice will be prepared.

### 2.3. Growth Conditions

Na_2_SeO_3_ and SeMet were added to PDA and MEA media, respectively. The concentrations of Na_2_SeO_3_ were 3 *μ*g/mL, 6 *μ*g/mL, and 10 *μ*g/mL, respectively. The concentrations of SeMet were 5 *μ*g/mL, 10 *μ*g/mL, and 20 *μ*g/mL. The media without supplementation of Na_2_SeO_3_ and SeMet served as the control. Using three-point inoculations, three grains of sclerotia of PT95 and Q1 strain were inoculated at a 9 cm Petri dish containing 25 mL of media. Plates were incubated in the dark at 25°C for 25 days.

### 2.4. Observation on the Growth and Development of Sclerotia

In the process of culture, observe the strain every day and record the time when the exudate and the sclerotia mature. Finally, take photos under natural light with a Nikon D90 digital camera.

### 2.5. Sclerotial Biomass, Carotenoid Extraction, and Determination

The sclerotia on the agar surfaces in Petri plates were separated and washed thoroughly with distilled water and dried at 50°C at constant weight to determine sclerotial biomass.

The extraction and determination of pigments were performed as a modified procedure described by Li et al. [14].

### 2.6. Determination of Ascorbic Acid in Sclerotia [16–17]

Add a little 2% oxalic acid to grind 0.4 g of sclerotium sample, mix with 2.6 mL of 0.03% MPA ice bath, centrifugate at 25000 r/min for 15 min, take the supernatant, add a little 5% trichloroacetic acid (TCA) solution, cool it in ice bath for 10 min, centrifugate at 150000 r/min for 5 min, discard precipitated protein, and keep supernatant in refrigerator at -80°C for one week.

Divide the supernatant into two parts (0.25 mL each), add 0.05 mL reagent A to one part, and add 0.05 mL reagent B to the other part. Use 0.25 mL 0.03% metaphosphoric acid (MPA) (5% TCA as solvent) as blank, keep the mixture and blank at 37°C for 3 h, take an ice bath slowly, add 0.375 mL 75% H_2_SO_4_ of ice, place at room temperature for 30 min, make the color change, and compare the color at 520 nm.

### 2.7. Determination of Oxidation Resistance

#### 2.7.1. Extraction of Antioxidants from Sclerotia

Accurately weigh 1 g of dry sclerotia; add 20 mL of 80% ethanol, respectively; extract it from a shaker at 50°C 150 r/min for 24 h; filter the filter paper and retain the filtrate; then reextract the filter residue with 20 mL of 80% ethanol; combine the filtrate twice; dilute it with 80% ethanol to 100 mL as the mother liquor; and dry and weigh the filter residue.

#### 2.7.2. Determination of Total Phenolic Content

The total phenolic content of the sample was determined by Folin cioncalteu colorimetry [18]. Add distilled water (2.8 mL) and Folin cioncalteu reagent (0.2 mL) into the sclerotium extract diluent (0.4 mL) or gallic acid (standard phenolic compound) diluent, mix them evenly, let them stand for 6-8 min, then add saturated Na_2_CO_3_ solution (0.6 mL), let them stand in the dark for 120 min at room temperature, and measure their OD value at 760 nm. The standard curve was drawn with 0.02 mg/mL, 0.04 mg/mL, 0.06 mg/mL, 0.08 mg/mL, and 0.10 mg/mL gallic acid diluent. The total phenolic content of different extracts is expressed as gallic acid equivalent.

#### 2.7.3. Reducing Power Assay

The reduction force of the sample was determined by the potassium ferricyanide reduction method [19].

Mix 1.0 mL of sample diluent of different concentrations with 2.5 mL of 50 mM phosphoric acid buffer (pH 7.0), add 2.5 mL of potassium ferricyanide solution (1%), shake the mixture well, keep the mixture at 50°C for 20 min, then cool it quickly, add 2.5 mL of TCA solution (10%) and centrifugate it for 10 min, take 1.25 mL of the supernatant, mix it with 1.25 mL of distilled water and 0.25 mL of FeCl_2_ solution (0.1%), and measure it at 700 nm absorbance.

The EC_50_ value of the sample is calculated according to the sample curve at 700 nm, and butylated hydroxytoluene (BHT) is used as the standard.

#### 2.7.4. DPPH Free Radical Scavenging Activity

Scavenging activity on 1,1-diphenyl-2-picrylhydrazyl (DPPH) free radical was assessed according to the method reported earlier with a slight modification [20]. Briefly, 0.2 mL of extracts was mixed with 0.8 mL of 80% ethanol. And then, 3 mL of DPPH solution was added. The mixture was shaken well and incubated at room temperature for 30 min, and the absorbance was measured at 517 nm in a spectrophotometer; EC_50_ value (mg/mL) is the effective concentration at which DPPH radicals were scavenged by 50% and was obtained by interpolation from linear regression analysis. BHT was used as the standard. The experiment was performed in triplicate and averaged.

#### 2.7.5. Determination of Chelating Ability of Ferrous Ion

The determination of the chelating ability of ferrous ions in the samples was evaluated according to the slightly modified literature [21] previously reported. Mix 1.6 mL of sample diluent of different concentrations with 1.6 mL of distilled water, add 0.4 mL of FeCl_2_ solution (0.5 mm), shake it well, then add 0.4 mL of FerroZine solution (0.5 mm), shake it vigorously for 1 min, place it at room temperature for 20 min, and measure its absorbance at 562 nm. The absorbance was measured in solvent instead of sample:

(1) $$\begin{array}{c} Ferrous\;ion\;chelating\;capacity\;\left(\%\right)=\times 100\%. \end{array}$$

The EC_50_ value of the sample is calculated according to the sample curve at 562 nm, and ethylene diamine tetraacetic acid (EDTA) is used as the standard.

### 2.8. Determination of Selenium Content [22]

#### 2.8.1. Standard Curve Drawing

Take 1.0 mL-5.0 mL of selenium standard solution (5 *μ*g/mL), respectively, and put it into the separating funnel, add 3 mL of o-phenylenediamine solution (0.1%) and fill it with distilled water to about 50 mL, mix it evenly, adjust the pH to 2.0 with formic acid (80%) and concentrated ammonia water, place the mixture in the dark at room temperature for 60 min, extract the mixture with 10 mL of toluene by shaking for 3 min, and then leave it for 8 min, discard the water layer, and turn the remaining solution transfer to a 50 mL volumetric flask and fix the volume with toluene, and compare the color at 335 nm.

#### 2.8.2. Determination of Selenium in Sclerotia

Accurately weigh 2 g of sclerotium, add 30 mL of mixed acid (*V*_HNO_3__ : *V*_H_2_SO_4__ = 4 : 1), cover the top plate, and digest it in the dark for 24 h. Heat and digest the solution after cold digestion for clarification, transfer it to a 50 mL volumetric flask after cooling, and fix the volume with water. Take 40 mL of the test solution and put it into the separating funnel, and the rest treatment is the same as above [23]:

(2) $$\begin{array}{c} Content\;\text{of }selenium\;\left(\mu g/g\right)=\frac{CV}{WN}, \end{array}$$

where *C* is the standard concentration of selenium obtained from the standard curve (*μ*g/mL), *V* is the sample volume from toluene extraction (mL), *W* is the sample weight (g), and *N* is the volume fraction of the sample used for determination after total constant volume.

### 2.9. Statistical Analysis

In the experiment, there are three repetitions, and the experimental results are expressed as the mean ± standard deviation. At the same time, the Duncan multiple comparison method [24] is used to compare the two averages.

## 3. Results

### 3.1. Effect of Selenium on the Differentiation of Q1

Adding Na_2_SeO_3_ and SeMet of different concentrations to PDA and MEA media, the results showed that there was almost no sclerotium formation on the surface of PDA medium, which indicated that PDA medium was not conducive to the differentiation of Q1 sclerotia after adding selenium. Compared with PDA medium, MEA medium supplemented with selenium was more conducive to the differentiation of Q1sclerotia.

It can be seen from Table 1 that when Na_2_SeO_3_ is added to the MEA medium, with the increase of Na_2_SeO_3_ concentration, the emergence time of exudate of Q1 strain is delayed by 1-2 days, the emergence time of sclerotia is delayed by 3-4 days, and the maturation time of sclerotia is delayed by 4-5 days. When SeMet was added to the MEA medium, with the increase of SeMet concentration, the emergence time of exudate of Q1 strain was delayed by 1-3 days, the emergence time of sclerotia was delayed by 4 days, and the maturation time of sclerotia was delayed by 6-7 days.

### 3.2. Effect of Selenium on the Biomass of Q1

Table 2 lists the sclerotium biomass of Q1 strain under different selenium-enriched culture conditions. It can be seen from the table that with the increase of selenium concentration, the sclerotium biomass of Q1 strain decreased correspondingly.

On the medium supplemented with Na_2_SeO_3_, there was a significant negative correlation between the sclerotium biomass of Q1 strain and selenium concentration (*R*_Q1_ = −0.939). When the concentration of Na_2_SeO_3_ in the medium was 10 *μ*g/mL, the sclerotium biomass of Q1 strain was the lowest, which was 0.09 g/plate, 0.39 times the control.

In addition to SeMet, there was a significant negative correlation between the sclerotium biomass of Q1 strain and selenium concentration (*R*_Q1_ = −0.985). When the SeMet concentration in the medium was 20 *μ*g/mL, the sclerotium biomass of Q1 strain was the smallest, which is 0.13 g/plate, 0.57 times the control.

### 3.3. Effect of Selenium on Carotenoid Content in Sclerotia of Q1 Strain

**Figure 1:**
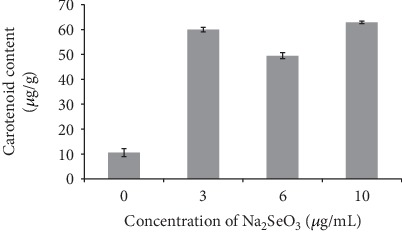
Effect of Na_2_SeO_3_ on carotenoid content in sclerotia of Q1 strain.

**Figure 2:**
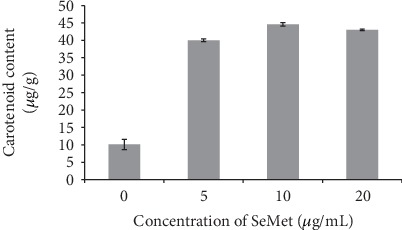
Effect of SeMet on carotenoid content in sclerotia of Q1 strain.

Figures 1 and 2 show the carotenoid content in the sclerotia of Q1 strain under different selenium-enriched culture conditions. It can be seen from the figure that the carotenoid content of the sclerotia of Q1 strain in different selenium-enriched culture conditions is significantly different from that in the control group (*P* < 0.05).

In the MEA medium supplemented with Na_2_SeO_3_, the content of carotenoids in the sclerotia of Q1 strain was positively correlated with the concentration of selenium (*R* = 0.752). When the concentration of Na_2_SeO_3_ in the medium was 10 *μ*g/mL, the content of carotenoids reached the maximum (62.9 *μ*g/g), which was 5.57 times the control.

In the MEA medium supplemented with SeMet, the content of carotenoids in the sclerotia of Q1 strain was positively correlated with selenium concentration (*R* = 0.689). When the concentration of SeMet in the medium was 10 *μ*g/mL, the content of carotenoids reached the maximum (44.6 *μ*g/g), which was 3.95 times the control.

### 3.4. Effect of Selenium on Ascorbic Acid Content in Sclerotia of Q1 Strain

Table 3 showed the effect of selenium on ascorbic acid content in sclerotia of *Penicillium thomii* Q1. It can be seen from the table that the AsA content of sclerotia grown in selenium-rich medium is higher than that of dehydroascorbic acid (DHA).

In the MEA medium, there was a positive correlation between the content of AsA and DHA in the sclerotia of Q1 strain and the concentration of Na_2_SeO_3_ (*R*_AsA_ = 0.683, *R*_DHA_ = 0.837) and a positive correlation between AsA/DHA and Na_2_SeO_3_ (*R* = 0.408). There was a negative correlation between AsA content and SeMet concentration (*R*_AsA_ = −0.450), a positive correlation between DHA content and SeMet concentration (*R*_DHA_ = 0.750), and a weak negative correlation between AsA/DHA and SeMet (*R* = −0.154).

In the MEA medium, there was a positive correlation between AsA content and Na_2_SeO_3_ concentration (*R*_AsA_ = 0.683), a significant negative correlation between DHA content and Na_2_SeO_3_ concentration (*R*_DHA_ = −0.999), and a positive correlation between AsA/DHA and Na_2_SeO_3_ (*R* = 0.790). There was a weak positive correlation between AsA content and SeMet concentration (*R*_AsA_ = 0.194), a certain positive correlation between DHA content and SeMet concentration (*R*_DHA_ = 0.365), and a weak positive correlation between AsA/DHA and SeMet (*R* = 0.033).

Compared with the control, Se-enriched culture can increase the content of AsA and DHA and AsA/DHA in the sclerotia of Q1strain. The content of AsA in sclerotia was higher than that of DHA in the same selenium-enriched culture condition.

### 3.5. Effect of Selenium on the Total Phenolic Content in the Sclerotia of Q1 Strain

**Figure 3:**
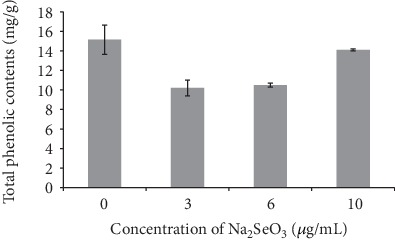
Effect of Na_2_SeO_3_ on the total phenolic content in the sclerotia of Q1 strain.

Figure 3 shows the effect of exogenous Na_2_SeO_3_ added to MEA medium on the total phenolic content in the sclerotia of *Penicillium thomii* Q1.

In the MEA medium, there was a significant negative correlation (*R* = −0.908) between the total phenolic content of the extract and the concentration of exogenous Na_2_SeO_3_. The total phenolic content of the control was the highest, reaching 15.15 mg/g. Under the different culture conditions, the total phenolic content of the sclerotia was MEA+Na_2_SeO_3_ 10 *μ*g/mL>MEA+Na_2_SeO_3_ 6 *μ*g/mL>MEA+Na_2_SeO_3_ 3 *μ*g/mL.

**Figure 4:**
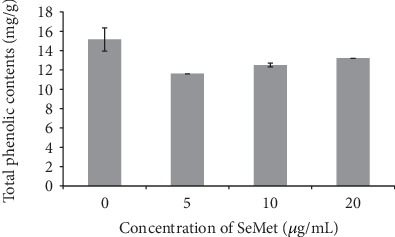
Effect of SeMet on the total phenolic content in the sclerotia of Q1 strain.

Figure 4 showed the effect of adding SeMet to the MEA medium on the total phenolic content in the sclerotia of *Penicillium thomii* Q1.

In the MEA medium, there was a significant negative correlation (*R* = −0.984) between the total phenolic content in the sclerotium extract of Q1 strain and the concentration of exogenous SeMet. The total phenolic content in the control was the highest (15.15 mg/g), and that in MEA+Na_2_SeO_3_ 20 *μ*g/mL>MEA+Na_2_SeO_3_ 10 *μ*g/mL>MEA+Na_2_SeO_3_ 5 *μ*g/mL.

In conclusion, the addition of Na_2_SeO_3_ and SeMet in the MEA medium was not conducive to the increase of total phenolic content in the sclerotia of Q1 strain.

### 3.6. Effect of Selenium on the Reduction Ability of Q1 Strain

Table 4 showed the effect of selenium-enriched culture conditions on the reduction ability of sclerotia of *Penicillium thomii* Q1. It can be seen from Table 5 that in the MEA medium, there is a certain negative correlation between EC_50_ value of reduction ability of sclerotium extract of Q1 strain and the concentration of exogenous Na_2_SeO_3_ (*R*_PT95_ = −0.793). The reduction ability under different culture conditions is MEA+Na_2_SeO_3_ 10 *μ*g/mL>MEA+Na_2_SeO_3_ 3 *μ*g/mL>MEA+Na_2_SeO_3_ 6 *μ*g/mL.

In the MEA medium, there was a negative correlation between EC_50_ value of the reduction ability of the extract of Q1 strain and the concentration of SeMet (*R* = −0.622). The reduction ability under different culture conditions was MEA+SeMet 10 *μ*g/mL>MEA+SeMet 5 *μ*g/mL>MEA+SeMet 20 *μ*g/mL.

In conclusion, when the MEA medium contains high concentration of exogenous Na_2_SeO_3_ (10 *μ*g/mL) and exogenous SeMet (20 *μ*g/mL), it is beneficial to improve the reducing power of the sclerotium extract of Q1 strain.

### 3.7. Effect of Selenium on DPPH Radical Scavenging Ability of Q1 Strain

Table 5 showed the effect of selenium-enriched culture conditions on DPPH free radical scavenging ability of sclerotia of *Penicillium thomii* Q1.

In the MEA medium, the EC_50_ value of DPPH free radical scavenging ability of the sclerotium extract of Q1 strain had a negative correlation with the concentration of exogenous Na_2_SeO_3_ (*R* = −0.230), and the DPPH free radical scavenging ability of the control was the largest (0.41 mg/mL). Under different culture conditions, the DPPH free radical scavenging ability was MEA+Na_2_SeO_3_ 10 *μ*g/mL>MEA+Na_2_SeO_3_ 3 *μ*g/mL>MEA+Na_2_SeO_3_ 6 *μ*g/mL.

In the MEA medium, there was a positive correlation between EC_50_ value of DPPH free radical scavenging ability and SeMet concentration (*R* = 0.241). The DPPH free radical scavenging ability of the control was the largest (0.47 mg/mL). The scavenging ability of DPPH free radical in different culture conditions was MEA+SeMet 20 *μ*g/mL>MEA+SeMet 5 *μ*g/mL>MEA+SeMet 10 *μ*g/mL.

These results showed that the addition of SeMet was not conducive to the improvement of DPPH free radical scavenging ability by Q1 strain, and the addition of Na_2_SeO_3_ (10 *μ*g/mL) with higher concentration was conducive to the improvement of DPPH free radical scavenging ability of Q1 strain, but the degree of improvement was not significant.

### 3.8. Effect of Selenium on the Chelating Ability of Ferrous Ions in the Sclerotia of Q1 Strain

Table 6 showed the effect of selenium-enriched culture conditions on the chelating ability of ferrous ions in the sclerotia of *Penicillium thomii* Q1.

It can be seen from Table 6 that in the MEA medium, there is a significant positive correlation (*R* = 0.954) between EC_50_ value of ferrous ion chelating ability of sclerotium extract of Q1 strain and the concentration of exogenous Na_2_SeO_3_. The ferrous ion chelating ability of the control is the largest (0.41 mg/mL). Under different culture conditions, the ferrous ion chelating ability was MEA+Na_2_SeO_3_ 3 *μ*g/mL>MEA+Na_2_SeO_3_ 6 *μ*g/mL>MEA+Na_2_SeO_3_ 10 *μ*g/mL.

In the MEA medium, EC_50_ value of ferrous ion chelating ability of the extract of Q1 strain was positively correlated with SeMet concentration (*R* = 0.981), and the ferrous ion chelating ability of the control was the largest (0.41 mg/mL). Under different culture conditions, the ferrous ion chelating ability was MEA+SeMet 5 *μ*g/mL>MEA+SeMet 10 *μ*g/mL>MEA+SeMet 20 *μ*g/mL.

The results showed that the addition of Na_2_SeO_3_ and SeMet was not conducive to the improvement of ferrous chelation ability by Q1 strain.

### 3.9. Effect of Selenium on Selenium Content of Sclerotia of Q1 Strain

**Figure 5:**
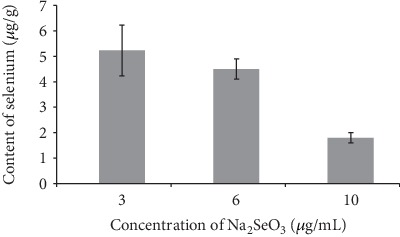
Effect of Na_2_SeO_3_ on selenium content in sclerotia of Q1 strain.

Figure 5 showed the effect of different concentration of Na_2_SeO_3_ added to the culture medium on selenium content in sclerotia of *Penicillium thomii* Q1.

It can be seen from Figure 5 that there was a weak positive correlation (*R* = 0.128) between the selenium content of the sclerotia of Q1 strain and the concentration of exogenous Na_2_SeO_3_ at the MEA medium, and it reached the maximum (5.23 *μ*g/g) when the concentration of Na_2_SeO_3_ was 3 *μ*g/mL.

**Figure 6:**
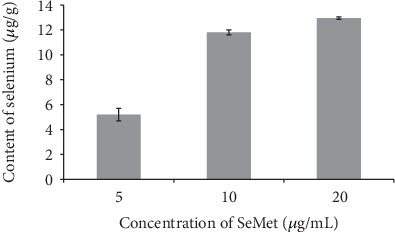
Effect of SeMet on selenium content in sclerotia of Q1 strain.

Figure 6 showed the effect of adding SeMet of different concentrations to the culture medium on the selenium content in the sclerotia of *Penicillium thomii* Q1.

It can be seen from Figure 6 that there was a significant positive correlation (*R* = 0.920) between the selenium content in the sclerotia of Q1 strain and the concentration of SeMet in the exogenous medium, and it reached the maximum (12.95 *μ*g/g) when the SeMet concentration is 20 *μ*g/mL.

The results showed that lower concentration of Na_2_SeO_3_ (3 *μ*g/mL) and higher concentration of SeMet (20 *μ*g/mL) in the MEA medium were beneficial to the increase of Se content in sclerotia of Q1 strain.

## 4. Discussion

Na_2_SeO_3_ is the most commonly used selenium-enriching agent [25], which is mainly used in the study of animals [26] and plants [27] and fungi [28] and large edible fungi [29]. However, Na_2_SeO_3_ is toxic, which limits its use. SeMet, as an organic selenium, is less toxic than Na_2_SeO_3_. However, the research of SeMet is not as extensive as that of Na_2_SeO_3_. At present, SeMet is mainly used as a selenium-enriching agent in animals [30] and plants [31], and its effect on yeast and large edible fungi in fungi has not been seen. In recent years, we have studied the effect of SeMet on the cultivation of *Pleurotus flavus* [32]. However, the selenium-enriched culture of Sclerotinia (mainly *Micrococcus*) fungi on Na_2_SeO_3_ and SeMet has not been reported.

Our previous experiments [13, 14] had shown that when the PT95 strain was grown under high oxidative stress condition, its sclerotial biomass was higher than that at low oxidative stress condition. Our experiments showed that the carotenoid, ascorbic acid, phenolic compounds, and selenium could be accumulated in the sclerotia of Q1 strain by adding different concentrations of inorganic and organic selenium in the MEA medium, and the content of carotenoid, ascorbic acid, and selenium in the sclerotia of Q1 strain could be increased to different degrees, but the content of total phenol could not be increased. Therefore, adding a certain concentration of selenium is beneficial to the increase of carotenoid and ascorbic acid content in sclerotia, but not to the increase of total phenolic content. So, we think that selenium may have some synergistic effect with carotenoids and ascorbic acid. These results indicated that ROS may induce antioxidant defense of Q1 strain by producing carotenoids instead of phenolic compounds in concentration levels related to the degree of oxidative stress. Mei et al. found that the structure of the fungal community was related to selenium content [33].

Selenium was found in *Cordyceps militaris*, *Penicillium expansum*, *Ganoderma lucidum*, *Saccharomyces cerevisiae*, etc. influencing the growth of fungi [33–36]. And selenium also affects antioxidant activity.

The antioxidant potential of the sclerotia could be attributed to its various characteristics. DPPH free radical scavenging activity and the reducing capacity of a compound may serve as a significant indicator of its potential antioxidant activity. In addition, when the concentration of Na_2_SeO_3_ and SeMet in the medium was 10 *μ*g/mL, the reducing power of Q1 sclerotium extract was improved. Except that the concentration of Na_2_SeO_3_ was 10 *μ*g/mL, the DPPH free radical scavenging ability of the extract from the sclerotia of strain Q1 could not be improved. Adding different concentrations of inorganic and organic selenium is not conducive to the improvement of the chelating ability of ferrous ions in Q1 sclerotium extract. Therefore, we think that the addition of selenium may change the proportion of various antioxidants in the antioxidant system, so that there are differences among the indicators.

## 5. Conclusions

Inorganic selenium and organic selenium were effectively added to the culture medium. Selenium could affect the differentiation and antioxidant activity of Q1 strain. The experimental results can provide a new research idea for the utilization and development of Penicillium sclerotium and selenium.

## Figures and Tables

**Table 1:** Effect of selenium on the differentiation of Q1 strain.

Culture conditions	Time of exudate initiation (days)	Time of sclerotial initiation (days)	Time of sclerotial maturation (days)
MEA	5^Aa^	6^A^	13^A^
MEA+Na_2_SeO_3_ 3 *μ*g/mL	6^B^	9^B^	17^B^
MEA+Na_2_SeO_3_ 6 *μ*g/mL	6^B^	9^B^	17^B^
MEA+Na_2_SeO_3_ 10 *μ*g/mL	7^C^	10^C^	18^C^
MEA+SeMet 5 *μ*g/mL	6^b^	10^B^	19^B^
MEA+SeMet 10 *μ*g/mL	8^C^	10^B^	20^C^
MEA+SeMet 20 *μ*g/mL	8^C^	10^B^	20^C^

^a^Superscript letters within a column indicate that values followed by the same letter did not differ significantly (*P* < 0.01) in Duncan's multiple range test.

**Table 2:** Effect of selenium on the sclerotium biomass of Q1 strain.

Culture conditions	Sclerotium biomass (mg/plate)
MEA	0.23 ± 0.02^C^
Na_2_SeO_3_ 3 *μ*g/mL	0.15 ± 0.01^B^
Na_2_SeO_3_ 6 *μ*g/mL	0.11 ± 0.03^B^
Na_2_SeO_3_ 10 *μ*g/mL	0.09 ± 0.01^A^
SeMet 5 *μ*g/mL	0.19 ± 0.04^B^
SeMet 10 *μ*g/mL	0.17 ± 0.01^B^
SeMet 20 *μ*g/mL	0.13 ± 0.02^A^

Superscript letters within a column indicate that values followed by the same letter did not differ significantly (*P* < 0.01) in Duncan's multiple range test.

**Table 3:** Effect of selenium on ascorbic acid content in sclerotia of Q1 strain.

Culture conditions	AsA content (*μ*g/g)	DHA content (*μ*g/g)	AsA/DHA
MEA	1.90 ± 0.26^A^	3.13 ± 0.32^DC^	0.61^A^
Na_2_SeO_3_ 3 *μ*g/mL	4.88 ± 0.62^C^	2.98 ± 0.31^C^	1.64^B^
Na_2_SeO_3_ 6 *μ*g/mL	4.75 ± 0.27^C^	2.82 ± 0.16^B^	1.69^BC^
Na_2_SeO_3_ 10 *μ*g/mL	4.59 ± 0.34^B^	2.64 ± 0.18^A^	1.74^D^
SeMet 5 *μ*g/mL	5.14 ± 0.66^D^	2.53 ± 0.43^A^	2.04^D^
SeMet 10 *μ*g/mL	4.08 ± 0.14^C^	3.05 ± 0.13^B^	1.34^C^
SeMet 20 *μ*g/mL	3.34 ± 0.68^B^	3.19 ± 0.37^C^	1.05^B^

Superscript letters within a column indicate that values followed by the same letter did not differ significantly (*P* < 0.01) in Duncan's multiple range test.

**Table 4:** Effect of selenium reduction ability on the of Q1 strain.

Culture conditions	EC_50_ (mg/mL)
MEA	1.49 ± 0.13^C^
Na_2_SeO_3_ 3 *μ*g/mL	0.88 ± 0.05^AB^
Na_2_SeO_3_ 6 *μ*g/mL	0.92 ± 0.03^B^
Na_2_SeO_3_ 10 *μ*g/mL	0.82 ± 0.02^A^
SeMet 5 *μ*g/mL	1.01 ± 0.08^A^
SeMet 10 *μ*g/mL	1.00 ± 0.06^A^
SeMet 20 *μ*g/mL	1.05 ± 0.06^AB^

Superscript letters within a column indicate that values followed by the same letter did not differ significantly (*P* < 0.01) in Duncan's multiple range test.

**Table 5:** Effect of selenium on DPPH radical scavenging ability of Q1 strain.

Culture conditions	EC_50_ (mg/mL)
MEA	0.47 ± 0.09^A^
Na_2_SeO_3_ 3 *μ*g/mL	0.59 ± 0.12^B^
Na_2_SeO_3_ 6 *μ*g/mL	0.64 ± 0.12^C^
Na_2_SeO_3_ 10 *μ*g/mL	0.41 ± 0.02^A^
SeMet 5 *μ*g/mL	0.86 ± 0.13^C^
SeMet 10 *μ*g/mL	0.88 ± 0.08^C^
SeMet 20 *μ*g/mL	0.66 ± 0.02^B^

Superscript letters within a column indicate that values followed by the same letter did not differ significantly (*P* < 0.01) in Duncan's multiple range test.

**Table 6:** Effect of selenium on the chelating ability of ferrous ions in the sclerotia of Q1 strain.

Culture conditions	EC_50_ (mg/mL)
MEA	0.41 ± 0.11^A^
Na_2_SeO_3_ 3 *μ*g/mL	1.23 ± 0.24^B^
Na_2_SeO_3_ 6 *μ*g/mL	1.25 ± 0.18^B^
Na_2_SeO_3_ 10 *μ*g/mL	2.67 ± 0.23^C^
SeMet 5 *μ*g/mL	0.94 ± 0.13^B^
SeMet 10 *μ*g/mL	1.15 ± 0.09^C^
SeMet 20 *μ*g/mL	2.65 ± 0.14^D^

Superscript letters within a column indicate that values followed by the same letter did not differ significantly (*P* < 0.01) in Duncan's multiple range test.

## Data Availability

The data used to support the findings of this study are available from the corresponding author upon request.
